# Supplementary material for: Association Between Renal Dysfunction and Lipid Ratios in Rural Black South Africans
Source: Int J Environ Res Public Health. 2025 Feb 21;22(3):324. doi: 10.3390/ijerph22030324 (PMC11942230; doi:10.3390/ijerph22030324)
Supplement: Supplementary file 1 [file ijerph-22-00324-s001.zip › ijerph-3475104-supplementary.pdf]

The association between renal dysfunction and Lipid/Lipid ratios amongst the rural black population of South Africa

Cairo B. Ntimana [1,*], Given R. Mashaba [1], Kagiso P. Seakamela[1], Peter M. Mphekgwana[2], Rathani Nemuramba[1], Katlego Mothapo[1], Joseph Tlouyamma[1], Solomon S.R Choma[4] and Eric Maimela[1]

[1] DIMAMO Population Health Research Centre, University of Limpopo, Sovenga St, Polokwane 0727, South Africa

[2] Research Administration and Development, University of Limpopo, Sovenga St, Polokwane 0727, South Africa

[3] Department of Public Health, University of Limpopo, Sovenga St, Polokwane 0727, South Africa

[4] Department of Pathology and Medical Sciences, University of Limpopo, Sovenga St, Polokwane 0727, South Africa

[*]Corresponding author:Email: cairo.ntimane@ul.ac.za

Table S1. Characteristics of participants by gender

	**Total**	**Men**	**Women**	**p-value**
Variables	N=1392	N= 427	N= 965	
Clinical characteristics				
Age (years)	52.07± 8.244	51.96±8.29	52.12±8.23	0.737
Waist circumference (cm)	89.84±16.05	80.63±11.60	93.91±16.07	<0.001
Central obesity n (%)	820 (58.7)	60 (14.0)	760 (54.4)	<0.001
BMI (kg/m^2)	27.54±8.05	21.71±4.04	30.55±7.89	<0.001
General obesity n(%)	495 (35.5)	14 (3.3)	482 (49.7)	<0.001
Current smoker n(%)	289 (20.7)	258 (60.3)	31 (3.2)	<0.001
Current alcohol consumption n(%)	387 (27.7)	257 (60.2)	130 (13.4)	<0.001

Systolic blood pressure (mmHg)	126.16±21.48	126.0±21.4	126.23±21.5	0.859
Diastolic blood pressure (mmHg)	80.68±12.91	78.66±12.77	81.57±12.88	<0.001
Hypertension n (%)	401 (28.7)	102 (23.8)	299 (30.8)	0.008
Serum lipids				
Total cholesterol (mmol/L)	4.14±1.15	3.94±1.03	4.22±1.18	<0.001
Triglycerides (mmol/L)	0.960 (1.34 - 0.71)	0.950 (1.33 - 0.69)	1.00 (2.70 - 0.51)	0.765
HDL-C (mmol/L)	1.20±0.40	1.25±0.48	1.18±0.36	0.003
LDL-C (mmol/L)	2.55±1.03	2.35±1.027	2.64±1.021	<0.001
TC/HDL-C ratio	3.71±1.37	3.52±1.67	3.79±1.20	0.003
TG/HDL-C ratio	0.863(1.28-0.56)	0.826(1.27-0.54)	0.873(1.31-0.59)	0.230
LDL-C/HDL-C ratio	2.34±1.69	2.60±2.21	1.04±2.40	0.143
Biochemical results				
Serum creatinine (μmol/L)	70.25±29.09	77.33±45.90	67.11±16.05	<0.001
eGFR (ml/min/1.73 m^2)	92.47±18.31	98.50±17.72	89.81±17.94	<0.001
ACR (mg/mmoL)	0.979 (2.49-0.48)	0.85 (2.37-0.38)	1.00 (2.70-0.51)	0.161
Fasting glucose (mmol/L)	5.24±2.34	5.05±2.08	5.33±2.45	0.030
Kidney dysfunction n (%)	163 (11.7)	36 (8.4)	127 (13.2)	0.011
